# Investigation on the multi-scale structure and digestibility of starch in sweet rice wine and its vinasse: Insights from indica and glutinous rice varieties

**DOI:** 10.1016/j.fochx.2025.102521

**Published:** 2025-05-09

**Authors:** Yongqiang Gong, JiaXing Hu, Ting Xie, Hongyu Mou, Shuzhi Xiao, Zihan Yao, Tao Yang

**Affiliations:** College of Food Science and Engineering, Central South University of Forestry and Technology, Changsha 410004, China

**Keywords:** Sweet rice wine, Vinasse, Starch, Multi-scale structure, Digestibility

## Abstract

Sweet rice wine is a traditional Chinese fermented beverage in which starch serves as the primary fermentation substrate. After sweet rice wine fermentation, low-molecular-weight starch remained in the fermentation mash (IRWS, indica sweet rice wine starch: 390.7 kDa; GRWS, glutinous sweet rice wine starch: 606.3 kDa), whereas higher-molecular-weight starch concentrated in the vinasse (IRVS, indica rice vinasse starch: 6954.9 kDa; GRVS, glutinous rice vinasse starch: 77,499.6 kDa). Compared with cooked rice starch, vinasse starches showed reduced digestibility, with resistant starch contents of IRVS and GRVS increasing from 25.48 % and 16.15 % to 30.88 % and 23.36 %, respectively. Structurally, GRVS retained a branched architecture similar to its native counterpart, whereas IRVS underwent significant structural changes, marked by an increase in long amylopectin branches, and a B + *V*-type crystallization formed based on high amylose content and starch retrogradation. Small-particle starch in the fermentation mash, IRWS and GRWS, exhibited B + *V*-type crystallization, high short-range order, elevated peak gelatinization temperatures (IRWS: 90.5 °C; GRWS: 101.3 °C), and strong resistance to enzymatic digestion. Both contained long-linear dextrin polymers with degrees of polymerization concentrated at 200–300 (IRWS) and 300–400 (GRWS). These results provide a theoretical basis for the quality improvement of sweet rice wine and the utilization of by-products.

## Highlights

• The multi-scale structure and digestibility of starch in sweet rice wine and vinasse were investigated.
• Short amylopectin chains were preferentially hydrolyzed during the fermentation.
• Vinasse starch retained many crystalline structures due to incomplete gelatinization and retrogradation.
• Small starch particles in sweet rice wine contained long linear dextrin.
• Distinct starch structure were observed between indica, glutinous sweet rice wines and their vinasses.

## 1 Introduction

Sweet rice wine, a traditional Chinese fermented beverage with a history spanning over 3000 years, is esteemed for its harmonious sensory profile, characterized by a sweet flavor, mild taste, delicate aroma, and its nutritionally rich composition (Yang, Zhong, Yang, Xu, & Yang, 2022). Classified into non-glutinous and glutinous rice varieties based on raw materials, this low-alcohol beverage derives its distinct properties from the interplay of enzymatic activity and microbial metabolism during production. The fermentation process not only enhances the sensory qualities of the sweet rice wine but also amplifies its nutritional value, aligning with the growing global emphasis on functional foods that confer health benefits beyond basic nutrition (Yan et al., 2024). Previous studies have proved that sweet rice wine is rich in nutrients, containing sugar, amino acids, organic acids, vitamins, polyphenols, γ-aminobutyric acid, active peptides and other nutrients (Chang et al., 2025; Guo et al., 2022; Shen et al., 2024). Sweet rice wine has a variety of health benefits, such as antioxidant, anti-aging, blood pressure lowering, cholesterol lowering, and immunomodulatory properties because of the presence of a variety of nutrients (Ma et al., 2024). Such functional enrichment aligns with broader trends in food science, where fermentation is leveraged to improve the bioavailability of nutrients and introduce health-enhancing metabolites.

To ensure product safety, flavor consistency, and bioactive functionality, modern sweet rice wine production predominantly employs pure-cultured *Rhizopus oryzae* as a starter culture. This controlled fermentation approach minimizes microbial contamination while standardizing enzymatic activity, thereby enhancing product quality and reproducibility (Yang, Zhong, Yang, Zhu, et al., 2022). The characteristic sweetness of the beverage arises from a sequential enzymatic cascade: during its growth phase, *Rhizopus oryzae* secretes α-amylase, which hydrolyzes α-1,4 glycosidic bonds in starch to generate dextrins, followed by glucoamylase-mediated *exo*-cleavage of these dextrins into glucose, maltose, and oligosaccharides (Salgaonkar et al., 2018). Concurrently, ambient yeasts (e.g., *Saccharomyces cerevisiae*), introduced via natural inoculation during open fermentation, metabolize monosaccharides through glycolysis, producing trace ethanol that contributes to the sweet rice wine's mild alcoholic undertones (Cai et al., 2018; Lai et al., 2019).

Due to the short fermentation cycle of sweet rice wine, starch conversion is incomplete, yielding two distinct by-products: (1) fermentation mash, which is abundant in incompletely hydrolyzed low-molecular-weight starch and dextrins; (2) vinasse, a solid by-product rich in unhydrolyzed high-molecular-weight starch (Lai et al., 2019). Dextrins in the fermentation mash has a significant impact on the unique taste and flavor of sweet rice wine. However, there were relatively few relevant literature reports. Zhang et al. (2017) demonstrated that incorporating resistant dextrins into sweet rice wine modulates its colloidal behavior, with reduced surface tension and increased viscosity of sweet rice wine. Furthermore, maltodextrins and linear dextrins exhibit molecular encapsulation capabilities due to their helical conformation, effectively stabilizing volatile aroma compounds and bioactive constituents within their hydrophobic cavities (Xiao et al., 2022). Therefore, dextrins in sweet rice wine has potential sustained-release substances for its flavor and functional substances. Compared with raw starch, the structure and functionality of vinasse starch change significantly following fermentation. Recent studies on the Baijiu fermentation of glutinous sorghum showed that long-term fermentation almost fully utilized the amylose part in the vinasse, causing a significant reduction in starch molecular weight and complete loss of granule integrity (Li, Huang, et al., 2024). For the nutritional value of vinasse starch, its digestibility cannot be ignored. In a related study, the starch retained in the vinasse of long-grain glutinous rice preserved a higher proportion of longer amylopectin branches and exhibited slow, controlled glucose release (Situ et al., 2019). Similarly, Zhang et al. (2016) found that the increase in A-type crystallinity and molecular helix in glutinous rice vinasse led to an increase in slow digestion. This proves that some vinasses have the potential to become slow-digesting foods.

Despite the critical role of starch and its derivatives in determining the physicochemical and sensory attributes of sweet rice wine and vinasse, the structural and functional characterization remains underexplored. Current research has predominantly centered on flavor profiling (volatile compound identification) and bioactive functionality (Lou et al., 2023; Yang, Liu, et al., 2021; Zhao et al., 2023). However, a significant knowledge gap persists regarding the molecular architecture of residual starch and dextrins in both the liquid phase (sweet rice wine) and solid residue (vinasse). To address these limitations, this study systematically investigates the microscopic structure, molecular weight distribution, whole-molecule size distribution, short-range order, crystalline structure, thermodynamic properties and in vitro digestion properties of starch in IRW, GRW, and their vinasse, using raw rice starch as a control. Considering the increasing popularity of low alcohol fermented wine, this research provides theoretical support for the further development of sweet rice wine and its vinasse products in the future.

## 2 Materials and methods

### 2.1 Materials

Indica rice was purchased from Shanghai Yihai Kerry Arawana Holdings Co., Ltd. (Shanghai, China). Glutinous rice was purchased from Anhui Yanzhifang Food Co., Ltd. (Hefei, China). Commercial *Rhizopus oryzae* starter was purchased from Angel Yeast Co., Ltd. (Yichang, China). Dispase (D195752, 50 U/mg) was obtained from Aladdin Biochemical Technology Co., Ltd. (Shanghai, China). This neutral protease shows optimal enzymatic activity at pH 6.0–8.0 and operates effectively within 30–50 °C. All other chemicals used were of analytical grade.

### 2.2 Preparation of sweet rice wine

The brewing method for sweet rice wine was adapted from the approach described by Yang, Zhong, Yang, Zhu, et al. (2022) with some modifications. Raw rice (100 g) was placed in a 500 mL beaker, to which 100 g of distilled water was added. Each beaker was then sterilized at 115 °C for 30 min. After cooling to 30 °C, a commercial *Rhizopus oryzae* starter was added at 0.4 % of the rice's weight and thoroughly mixed. The beakers were incubated at 30 °C for 48 h, after which the sweet rice wine and vinasse were separated using 40-mesh sieve for subsequent analysis.

### 2.3 Preparation of starch in sweet rice wine, vinasse and raw rice

Indica starch and glutinous rice starch were extracted by using alkali extraction, following the method reported by Bian et al. (2022) The vinasse was crushed with water using a high-speed disperser (MJ-LZ25Easy121, Midea Group Co., Ltd., Foshan, China). The pH of both the vinasse suspension and sweet rice wine was adjusted to 7.0 using 0.1 mol/L NaOH solution. Adding a triploid volume of ethanol to extract starch, then after centrifuging at 4000 r/min for 10 min, the precipitate was washed with ethanol, twice. After centrifugation at 4000 r/min for 10 min, the precipitate was washed twice with ethanol. Lyophilized samples were crushed and passed through a 100-mesh sieve.

### 2.4 Chemical analysis of sweet rice wine

The sweet rice wine was centrifuged at 4000 r/min for 10 min, and the supernatant was collected to determine the reducing sugar and total sugar content using the DNS method (Di et al., 2023). The total acid content was determined according to the Chinese standard (GB 12456–2021). The sweet rice wine was diluted ten times with distilled water, and 25 mL of the diluent with four drops of phenolphthalein solution (10 g L^−1^) were titrated with 0.1 mol/L NaOH standard solution until the red end point and NaOH consumption was recorded. 100 mL sweet rice wine was centrifuged at 4000 r/min, and after freeze-drying, the content of insoluble glucan in 100 mL of sweet rice wine was determined using the starch content assay kit. The total acid content was calculated according to the formula provided in GB 12456–2021. Alcohol content was determined according to the method pro*v*ided by Yang, Zhong, Yang, Xu, and Yang (2022).

### 2.5 Amylose content

The amylose content was determined as the method described by Deng et al. (2024). Starch (0.1 g) was dispersed in 1 mL of absolute alcohol and 9 mL of 1 mol/L NaOH in a boiling water bath for 30 min. The dispersion was diluted to 50 mL with distilled water. A 2.5 mL aliquot was taken, and 0.1 mol/L acetic acid was added to adjust the pH to neutral. Subsequently, 2 mL of iodine solution (0.2 % I₂ and 2 % KI, *w*/*v*) was added, and the solution was diluted to 100 mL with distilled water. The mixture was allowed to stand at room temperature for 15 min. The absorbance was measured at 620 nm using a UV–Vis spectrophotometer (756PC, Shanghai Sunny Hengping Scientific Instrument Co., Ltd., Shanghai, China). The amylose content was calculated using a standard curve and expressed as a percentage of the sample's dry weight.

### 2.6 Morphological

A scanning electron microscope (SEM, S-4800, Hitachi, Japan) was used to analyze the morphological characteristics of the samples. The granular samples were thinly spread on circular aluminum stubs using conductive adhesive and coated with a thin layer of gold. All samples were observed at an accelerating voltage of 15 kV with a magnification of 5000 and 10,000 × times.

### 2.7 Molecular weight distribution analyses

Starch (5 mg) was thoroughly mixed with 5 mL DMSO solution containing lithium bromide (0.5 % *w*/w) (DMSO/LiBr) and heated at 80 °C using a thermomixer for 3 h. The homogeneity and molecular weight of various fractions were measured using. SEC-MALLS-RI. The molecular weight (Mw) and polydispersity index (PDI) of various fractions in DMSO/LiBr (0.5 % w/w) solution weremeasured on a DAWN HELEOS-II laser photometer (He—Ne laser, λ = 663.7 nm, Wyatt Technology Co., Santa Barbara, CA, USA) equipped with Three tandem columns (300 × 8 mm, Shodex OH-pak SB-805, 804 and 803; Showa Denko K.K., Tokyo, Japan) which was held at 60 °C using a model column heater. Technical support is provided by Sanshu Biotech. Co., Ltd. The flow rate is 0.3 mL/min. The dn/dc value of the fractions in DMSO solution was determined to be 0.07 mL/g. Data were acquired and processed using ASTRA6.1 (Wyatt Technology). Quantified data were output into excel format.

### 2.8 Whole-molecule size distribution analyses

Starch (5 mg) was dissolved in 0.9 mL water in a boiling water bath for 15 min. Sodium azide solution (5 mL, 40 mg/mL), acetate buffer (0.1 mL, 0.1 mol/L, pH 3.5, prepared using 0.1 mol/L acetic acid solution and 0.1 mol/L sodium acetate solution), and isoamylase (10 uL, 1400 U) were added to the starch dispersion, and the mixture was incubated in a water bath at 37 °C for 3 h. The debranched starch was precipitated with 5 mL absolute ethanol, centrifuged at 4000 r/min for 10 min, and then redissolved in 1 mL DMSO/LiBr solution for 2 h at 80 °C in a thermomixer with shaking at 350 rpm. The homogeneity and molecular weight of various fractions were measured using SEC-RI. The molecular weight distribution of debranched starch was analyzed using a differential refractive index detector (Optilab T-rEX, Wyatt Technology Co., Santa Barbara, CA, USA) equipped with two tandem columns (300 × 7.5 mm, Plael 10 um MIXED-B and Plgel 5um MIXED-D; Agilent Technologies Inc., CA, USA) which was held at 80 °C using a model column heater. Technical support is provided by Sanshu Biotech. Co., Ltd. The flow rate was set at 0.8 mL/min, and standard dextrans with known molecular weights (342; 3650; 21,000; 131,400; 610,500; 821,700; 3,755,000) were employed for column calibration. Data were acquired and processed using ASTRA6.1 (Wyatt Technology). Quantified data were output into excel format.

### 2.9 Fourier transform infrared spectroscopy (FT-IR)

FT-IR spectra were recorded using an FT-IR spectrometer (Nicolet iS10, Thermo Fisher Scientific Co. Ltd., Waltham, Massachusetts, USA) with a scanning range of 4000–400 cm^−1^, a resolution of 4 cm^−1^, and 64 scans. The 1100–900 cm^−1^ region was deconvoluted to analyze the short-range ordered structures of the samples (Deng et al., 2024).

### 2.10 X-ray diffractometry (XRD)

The crystal structure of the sample was determined using an X-ray diffractometer (D2 PHASER, Bruker AXS Co. Ltd., Karlsruhe, Germany). Diffraction patterns were recorded at a scanning rate of 2°/min over a 5°-40° angular range (Lu et al., 2019).

### 2.11 Differential scanning calorimetry (DSC)

The onset temperature (To), peak temperature (Tp), conclusion temperature (Tc), and gelatinization enthalpy (ΔH) were determined using a differential scanning calorimeter (DSC8000, PerkinElmer Instruments Co., Ltd., Shanghai, China). A 2 mg starch sample was placed in a sealed aluminum pan with 6 μL of deionized water, equilibrated at 25 °C for 24 h, and then heated from 30 °C to 130 °C at a rate of 10 °C/min. An empty pan served as the reference (Qiao et al., 2024).

### 2.12 In vitro digestion properties

According to the method of Englyst et al. (1992) with some modification. Porcine pancreatic α-amylase (75 mg, 9 U/mg) was dissolved in 20 mL 0.2 mol/L sodium acetate buffer (pH 5.5) and stirred magnetically for 30 min to ensure complete dissolution. The porcine pancreas α amylase solution was centrifuged at 5000 r/min for 5 min, and the supernatant was mixed with starch glucosidase (7 mg, 100 U/mg). Samples (100 mg) and 10 mL sodium acetate buffer (0.2 mo1/L, pH 5.5) were dispersed in 50 mL centrifuge tubes and shaken at 120 r/min for 20 min in a thermostatic water bath at 37 °C. Then 0.75 mL of the mixed enzyme solution was added. The reaction times were 20 min and 120 min, respectively. The reaction was stopped by adding 5 mL absolute ethanol and centrifuged. The glucose concentration of the supernatant was measured using a GOPOD kit. The contents of rapidly digestible starch (RDS), slowly digestible starch (SDS) and resistant starch (RS) were calculated by the following (1), (2), (3):

(1) $$\mathrm{RDS}\;\left(\%\right)=\frac{\left(G20-G0\right)\times 0.9}{M}\times 100$$

(2) $$\begin{array}{c} \mathrm{SDS}\;\left(\%\right)=\frac{\left(G120-G20\right)\times 0.9}{M}\times 100 \end{array}$$

(3) $$\begin{array}{c} \mathrm{RS}\;\left(\%\right)=100-\mathrm{RDS}-\mathrm{SDS} \end{array}$$

M represents the total starch mass, mg; G20 and G120 are the glucose content released after 20 min and 120 min, mg, respectively.

### 2.13 Statistical analysis

All numerical results are the average of at least three independent replicates. Analysis of variance using Duncan's test and Pearson's bivariate correlation analysis were evaluated using SPSS 19.0 (SPSS Inc., Chicago, USA). Origin2021 software (OringinLab Corporation, Northampton, USA) was used for plotting. Values in the same column with different superscripts are significantly different (*p* < 0.05).

## 3 Results and discussion

### 3.1 Chemical analysis of two rice wines

The results of the chemical analysis of the sweet rice wine are shown in Table 1. The sweet rice wine after vinasse removal possessed more total soluble sugars in GRW (53.52 g L^−1^) than IRW (61.61 g L^−1^). In addition, GRW (37.26 g L^−1^) possessed higher reducing sugars relative to IRW (26.21 g L^−1^). The cooked GRS with amylopectin as the main content is easier to be hydrolyzed by amylase than the cooked IRS (Zhou et al., 2025), which will be further confirmed in the subsequent experiments. It is worth noting that the total sugars contained some soluble oligosaccharides without reducing properties in addition to reducing sugars, with IRW (27.31 g L^−1^) slightly higher than GRW (24.35 g L^−1^). In contrast, the insoluble glucan in IRW (34.80 g L^−1^) was 3.8 times higher than that in GRW (9.21 g L^−1^). This result can be explained by the higher resistance to enzymatic degradation exhibited by the small-molecule starch retained in IRW, which leads to a greater retention of insoluble glucans in the fermentation mash. In addition, the presence of organic acids as well as a small amount of alcohol in the sweet rice wine was attributed to the small amount of lactic acid bacteria and yeasts enriched from the air present in the sweet rice wine utilizing sugar for fermentation to produce organic acids and ethanol, respectively (SantˈAna & Lemos Junior, 2024).

**Table 1:** Fermentation characteristics of IRW and GRW.

Samples	Reducing sugar (g L^−1^)	Total soluble sugar (g L^−1^)	Insoluble glucan (g L^−1^)	Total acid (g L^−1^) (calculated as lactic acid)	Alcohol (%vol)
IRW	26.21 ± 1.36	53.52 ± 2.11	34.80 ± 1.69	4.65 ± 0.24	1.53 ± 0.12
GRW	37.26 ± 1.61	61.61 ± 1.73	9.21 ± 0.75	4.89 ± 0.31	2.14 ± 0.19

### 3.2 Amylose content

As mentioned in Section 3.1, a large amount of small molecular starch produced by enzymatic hydrolysis was retained in IRW and GRW, and the main residual starch was retained in the vinasse. To elucidate the structural basis of these residual glucans, the molecular architecture should be further characterized, focusing on the linear (amylose) and branched (amylopectin) fractions. Table 2 presents the amylose content of small-molecule starch extracted from IRW and GRW, as well as that of their corresponding vinasse and raw rice starch. The amylose contents of IRS and GRS were 18.63 % and 0.94 %, respectively, aligning with previous findings (Wang et al., 2017). Notably, significant disparities emerged in amylose distribution between sweet rice wine and vinasse. Specifically, IRWS exhibited a reduced amylose content (16.80 %) compared to IRS, whereas IRVS showed a marked increase (19.82 %). This divergence can be attributed to the distinct physicochemical behaviors of amylose and amylopectin during fermentation. During the fermentation of cooked indica rice, starch retrogradation occurs concurrently. Compared to amylopectin, amylose exhibits a greater tendency to recrystallize post-gelatinization, forming structures with enhanced resistance to enzymatic hydrolysis (Li et al., 2022). In contrast, GRWS and GRVS displayed amylose contents of 7.07 % and 2.83 %, respectively, both exceeding the baseline level in GRS (0.94 %). Similar findings were also reported by Lai et al. (2019), who confirmed the enrichment of amylose in indica rice and glutinous rice vinasses during a 48-h fermentation process mediated by *Aspergillus oryzae*. Compared to GRS, GRWS exhibited a significantly higher amylose content. Partial debranching of amylopectin generated additional linear fragments, leading to a marked increase in short-chain amylose (Lu et al., 2024). Furthermore, the preferential hydrolysis of amylopectin in the fermentation broth further facilitated the accumulation of amylose. These findings underscore the critical role of enzymatic selectivity in reshaping starch architecture during fermentation.

**Table 2:** Amylose content, molecular weight, gyration radius and R_1047/1022 cm_^−1^ of starch in raw rice, sweet rice wine and vinasse.

Samlples	Amylose content	Mw (kDa)	PDI (Mw/Mn)	Rz (nm)	R_1047/1022 cm_^−1^
IRS	18.63 ± 0.21^b^	175,641.80 ± 0.30^b^	1.88 ± 0.38^e^	214.69 ± 0.10^b^	0.622 ± 0.009 ^c^
GRS	0.94 ± 0.11^f^	263,679.72 ± 0.42^a^	1.47 ± 0.50^e^	267.16 ± 0.09^a^	0.619 ± 0.001 ^c^
IRWS	16.80 ± 0.13^c^	390.74 ± 0.17^f^	5.73 ± 0.28^c^	117.31 ± 0.16^e^	0.868 ± 0.002 ^a^
GRWS	7.07 ± 0.15^d^	606.26 ± 0.30^e^	4.51 ± 0.59^d^	58.51 ± 1.05^f^	0.804 ± 0.003 ^b^
IRVS	19.82 ± 0.22 ^a^	6954.86 ± 0.21^d^	8.96 ± 0.30^b^	129.54 ± 0.18^d^	0.630 ± 0.004 ^c^
GRVS	2.83 ± 0.08^e^	77,499.56 ± 0.31^c^	10.05 ± 0.38^a^	201.60 ± 0.12^c^	0.586 ± 0.007 ^d^

A Data with different alphabets in the same column were different with statistical significance (p < 0.05). Mean values are given with standard deviations (*n* = 3).

### 3.3 Morphology

**Fig. 1:**
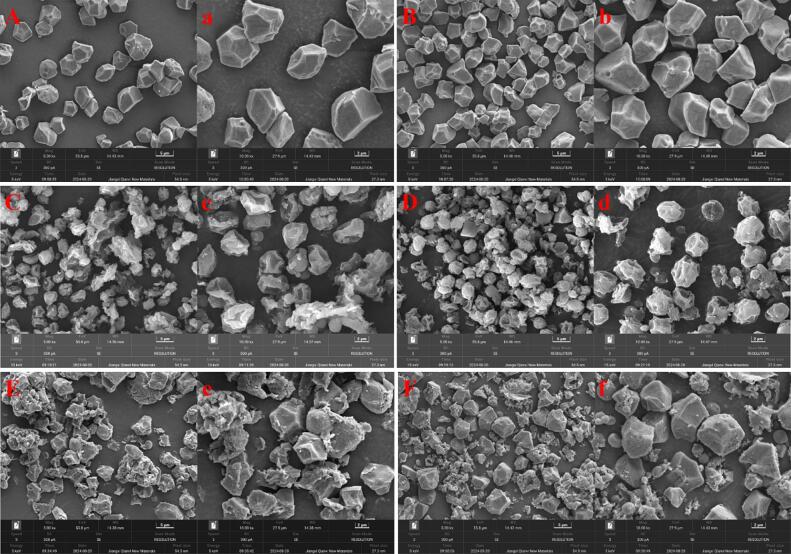
SEM images of starch granules in raw rice, sweet rice wine and vinasse. IRS (A, a), GRS (B, b), IRWS (C, c), GRWS (D, d), IRVS (E, e), GRVS (F, f).

Scanning electron microscopy (SEM) analysis revealed distinct morphological differences among starch granules in sweet rice wine, vinasse, and raw rice starch (Fig. 1). IRS and GRS granules exhibited characteristic polygonal morphologies with smooth surfaces and uniform particle sizes, consistent with the reported by Wang et al. (2022). IRWS and GRWS displayed pronounced surface alterations, containing collapsed granular structure and irregular pitting. These morphological disruptions align with the “inside-out” enzymatic hydrolysis mechanism proposed by Ma et al. (2020). Notably, spherical starch granules of varying sizes were observed in both IRWS and GRWS. These granules primarily represent intermediates of enzymatic starch hydrolysis, exhibiting morphological similarities to the maltodextrin structures described by Raj et al. (2024). In cooked rice, complete gelatinization was not achieved, and thus, in vinasse, it could be observed that a portion of the starch retains its original granular morphology, while another portion was consisted of small starch fragments produced by amylase hydrolysis. More fragments were observed in GRVS compared to IRVS, proving a more drastic amylase hydrolysis of starch in cooked glutinous rice. It is related to the high amylopectin content of GRS (Zhou et al., 2025).

### 3.4 Molecular weight distributions

Table 2 demonstrates significant differences in the molecular weight (Mw) of starch among raw rice, sweet rice wine, and vinasse. Starch in sweet rice wine and vinasse displayed markedly reduced Mw compared raw rice starch, indicative of extensive enzymatic depolymerization during fermentation. During fermentation, amylase hydrolyzed starch into smaller molecules, which were released into the fermentation mash for further degradation, while larger Mw starch was retained in the vinasse (Li, Long, et al., 2024). GRS (263,679.72 kDa) exhibited a significantly higher Mw than IRS (175,641.80 kDa), due to its amylopectin-dominant composition (Kowittaya & Lumdubwong, 2014). This also explains the narrower polydispersity index (PDI) of GRS (1.47) compared to IRS (1.88), as amylopectin's homogeneous branching patterns yield more uniform Mw distributions.

**Fig. 2:**
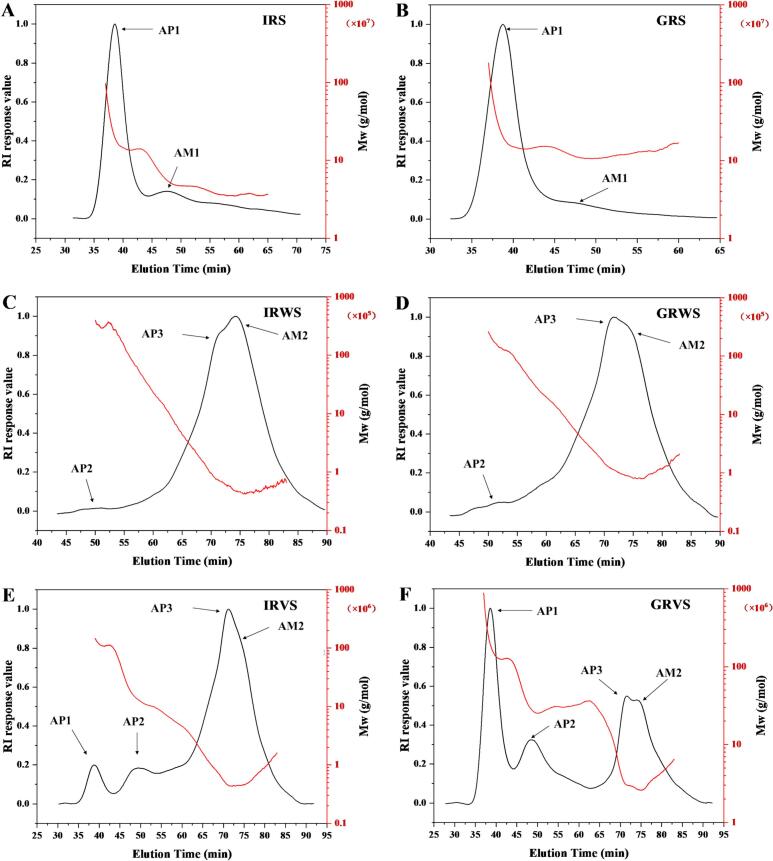
Whole-molecule size distributions of starch in raw rice, sweet rice wine and vinasse.

Further insights were derived from molar mass curves and refractive index curves are shown in Fig. 2A-F. Larger starch molecules eluted earlier, as exemplified by the prominent AP1 peak (35–40 min) corresponding to highly polymerized amylopectin in IRS and GRS (Gu et al., 2023). Conversely, the peak observed at 45–50 min (AM1) was associated with highly polymerized amylose (He et al., 2023). As the starch in sweet rice wine, a low-intensity peak (AP2) was detected at 45–50 min, suggesting the presence of a small amount of amylopectin. Two distinct peaks were observed in both IRWS and GRWS between 65 and 85 min, corresponding to branched dextrin (AP3) and long linear dextrin (AM2) (Londoño-Hernández et al., 2017). The Mw of IRWS and GRWS were 390.74 kDa and 606.26 kDa, respectively, attributed to the higher content of small amylopectin (AP2) and branched dextrin (AP3) in GRWS. Notably, the degree of polymerization (DP) of long linear dextrins in IRWS ranged mainly from 200 to 300, while in GRWS, it ranged from 300 to 400, with no low-molecular-weight linear dextrins detected. This observation may result from the preferential hydrolysis of low-molecular-weight linear dextrins by α-amylase and glucosidase (Li, Huang, et al., 2024). Four distinct peaks were observed during elution in Fig. 2E-F, corresponding to highly polymerized amylopectin (AP1), small amylopectin (AP2), branched dextrin (AP3), and long linear dextrin (AM2). This distribution showed a high PDI, indicating an uneven Mw distribution of vinasse starch. The AP1 peak in both IRVS and GRVS weakened significantly compared to its raw rice starch, while new peaks (AP3, AM2) appeared. The Mw of amylopectin and amylose with high DP decreased substantially after fermentation. Compared with sweet rice wine, branched (AP3) and linear dextrins (AM2) with higher DP were retained in the vinasse. The AP1 peak in GRVS remained the most prominent, and its Mw (77,499.56 kDa) was significantly higher than IRWS (6954.86 kDa), which was related to the large Mw of GRS.

### 3.5 Whole-molecule size distributions

**Fig. 3:**
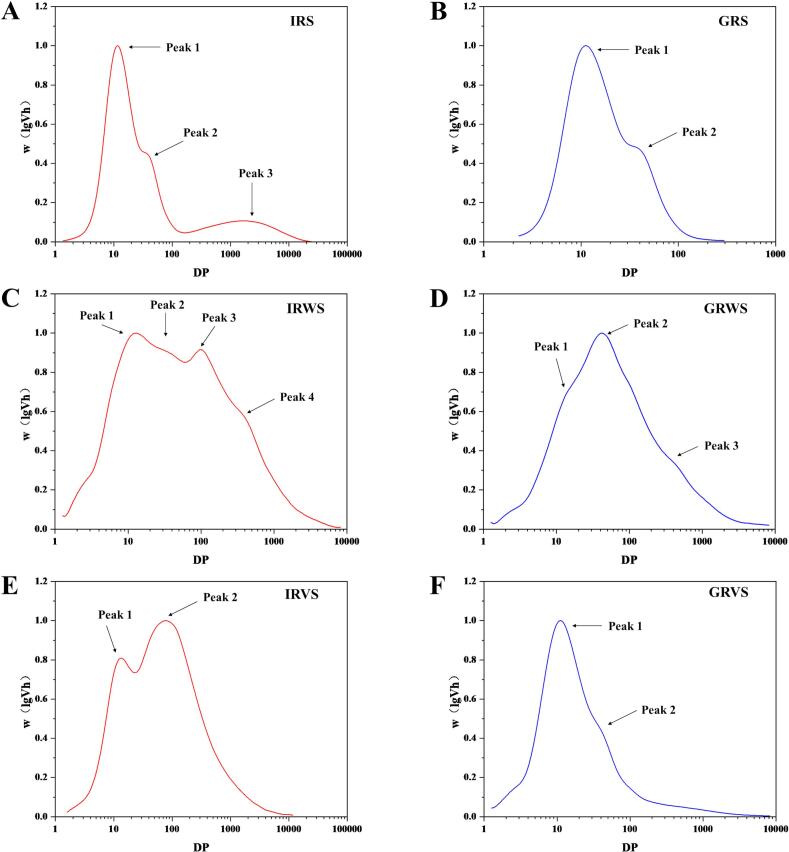
Whole-molecule size distributions of starch in raw rice, sweet rice wine and vinasse.

The whole-molecule size distributions of starch in raw rice, sweet rice wine, and vinasse are shown in Fig. 3A-F, where starch chain length was represented by the DP. According to previous studies (Li, Long, et al., 2024), amylopectin and amylose typically cover the DP ≤ 100 and DP > 100 ranges, respectively. As shown in Fig. 3A-B, both IRS and GRS exhibited a bimodal distribution, with amylopectin characterized by short amylopectin branches, indicating a higher branching density. A distinct peak at DP > 100 was observed in IRS, attributed to the presence of amylose. In contrast, GRS contains less amylose, and no peak was observed at DP > 100.

As shown in Fig. 3C, Peak 1 (DP ≈ 10) exhibited a noticeable peak, related to the release of small branched dextrins during starch hydrolysis. Peak 2 (DP 10–100) in IRWS and GRWS exhibited greater intensity compared to raw rice starch (DP 10–100), suggesting that short amylopectin branches are more susceptible to enzymatic hydrolysis than long amylopectin branches. This finding aligns with the observations of Situ et al. (2019), who reported an increase in long amylopectin branches in vinasse starch after fermenting raw rice with yeast, which was attributed to glucoamylase exhibited significantly lower specific activity toward α-1,6-glycosidic linkage compared to α-1,4-glycosidic linkage. Peak 3 (DP ≈ 100) in IRWS represented a mixture of long amylopectin branches and linear dextrins. In addition, peak 4 (DP 200–300) in IRWS and peak 3 (DP 300–400) in GRWS correspond to Mw linear dextrins with higher DP, which was consistent with the Mw distribution results. Vinasse starch exhibited a bimodal distribution, as shown in Fig. 3E-F. For IRVS, Peak 1 represented short amylopectin branches, while Peak 2 was divided into two regions: the portion with DP ≤ 100 predominantly consists of long amylopectin branches, and the portion with DP > 100 primarily consists of amylose. A greater amount of amylose was retained in the vinasse due to the recrystallization of amylose in IRS after gelatinization, resulting in the formation of more structures resistant to enzymatic degradation. This observation was consistent with the measured amylose content. This phenomenon was also observed in GRVS, where amylopectin retained a structure similar to GRS, but with an increased amylose content.

### 3.6 FTIR spectra analys

**Fig. 4:**
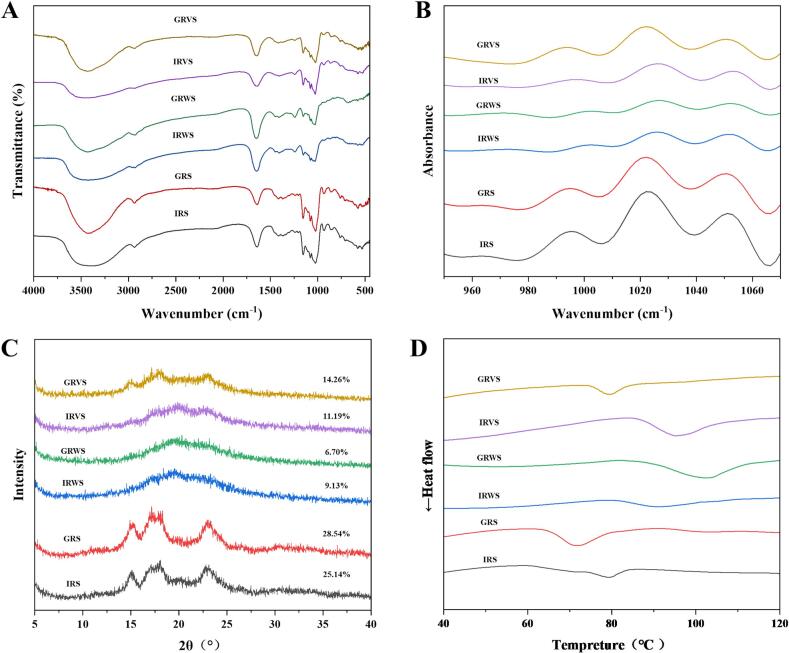
FT-IR (4000–500 cm^−1^) (A), deconvoluted FT-IR (950–1070 cm^−1^) (B), XRD (C), DSC-curve (D) spectra of starch in raw rice, sweet rice wine and vinasse.

Fourier-transform infrared (FT-IR) spectroscopy and deconvoluted spectral analysis provided critical insights into the hydrogen bonding and short-range molecular order of starch in raw rice, sweet rice wine, and vinasse (Fig. 4A–B). The absorption band observed at 3000–3500 cm^−1^ corresponded to the O—H stretching vibrations in starch, with the width of the peak indicating the extent of inter- and intramolecular hydrogen bonding (Qiu et al., 2016). Compared to raw rice starch and vinasse starch, the starch in sweet rice wine exhibited the highest wavenumber for hydroxyl stretching in the 3000–3500 cm^−1^ region. This shift toward higher wavenumbers signifies intensified hydrogen bonding interactions (Shao et al., 2025), likely arising from the reorganization of long linear dextrins and long amylopectin branches into tightly packed helical structures during fermentation. Furthermore, the 1047/1022 cm^−1^ ratio (R_1047/1022 cm_^−1^) in the deconvoluted spectra is often employed to assess alterations in short-range molecular order, where 1047 cm^−1^ and 1022 cm^−1^ bands represent ordered crystalline domains and amorphous regions, respectively (Duyen et al., 2022; Lin et al., 2024). As shown in Table 2, the R_1047/1022 cm_^−1^ values for IRWS and GRWS (0.868 and 0.804) were significantly higher than raw rice starch. Such a phenomenon can be attributed to long linear dextrins and long amylopectin branches formed during fermentation, forming a large number of single and double helix structures (Chi, Guo, et al., 2023; Chi, Zhou, et al., 2023). The observed decrease in the R_1047/1022 cm_^−1^ of GR*V*S relative to GRS can be attributed to the disruption of the crystalline structure induced by gelatinization (Liang et al., 2025). In contrast, IRVS showed no significant difference in this ratio from IRS, although the ordered structure was lost after gelatinization, the retention of more amylose in IRVS compensates for this change.

### 3.7 Crystalline structure

Fig. 4C shows XRD patterns and RC values for starch in raw rice, sweet rice wine and vinasse. The crystalline structure of raw rice starch was arranged in A-type, with diffraction peaks located at 15°, 17°, 18° and 23.5° (2θ) (Zhang et al., 2025). The crystallinity of GRS (28.54 %) was higher than IRS (25.14 %), since densely branched clusters of amylopectin facilitate the formation of well-ordered microcrystalline domains (Wang et al., 2022). IRWS and GRWS exhibited B + *V*-type crystallization with characteristic peaks at 17°, 20° and 23°. B-type crystallinity is formed by starch retrogradation, whereas V-type crystallinity is associated with amylose (Lin et al., 2024). The above analysis had proved that both IRWS and GRWS containd many single helical structures composed of long linear dextrins. IRWS exhibited a higher crystallinity (9.13 %) than GRWS (6.70 %), likely due to the higher content of long linear dextrins in IRWS, which favored the formation of crystalline structures (Liu et al., 2022). The crystallinities of IRVS and GRVS were 11.19 % and 14.26 % respectively, which was related to the fact that all starch could not be completely gelatinized during cooking. This was reflected by SEM that some starches in IRVS and GRVS still retained the original starch structure. In addition, starch retrogradation during fermentation can also promote the formation of crystalline structures (Gong et al., 2024). GRS demonstrated higher anti-gelatinization ability, resulting in GRVS retaining a higher proportion of crystalline structures and exhibiting an A-type crystalline pattern with attenuated characteristic peaks. In contrast, IRVS retained a greater amylose content and displayed a B + V-type crystallization. Although starch in sweet rice wine exhibited higher short-range order than in vinasse, incomplete gelatinization of the rice resulted greater crystallinity in vinasse.

### 3.8 Thermal properties

The thermodynamic behavior of starch in sweet rice wine, vinasse and raw rice was investigated using differential scanning calorimetry (DSC), with gelatinization curves illustrated in Fig. 4D and thermal parameters summarized in Table 3. IRS exhibited a peak temperature (T_P_) of 79.68 °C and enthalpy (∆H) of 4.07 J/g, while GRS showed a T_P_ of 71.59 °C and ∆H of 10.63 J/g. This is attributed to the easy gelatinization and high crystallinity of GRS, among which ΔH represents the energy required to break the starch helical structure and melt the crystals (Peng et al., 2021). Both IRWS and GRWS exhibited higher gelatinization temperatures (79.82–100.19 °C and 89.32–111.81 °C, respectively), attributed to the recrystallization of long linear dextrins. Notably, GRWS showed a higher T_P_ (101.3 °C) and ∆H (12.64 J/g) than IRWS (T_P_: 90.49 °C, ∆H: 5.83 J/g). The study by Chi, Zhou, et al. (2023) revealed a significant positive correlation between thermal stability and the chain length of linear dextrins. The above results showed that GRWS exhibited a higher DP in its long linear dextrins compared to IRWS, thereby accounting for its higher thermal stability. In addition, the double helix structure formed by the long amylopectin branches of GRWS also played an important role in the enhancement of thermal stability (Peng et al., 2021). Based on the same theory, IRVS, which had more amylose and longer amylopectin chains relative to GRVS, exhibited a higher gelatinization temperature and ΔH. These research findings indicate that the products of starch fermentation in sweet wine have led to the formation of numerous heat-resistant structures.

**Table 3:** Thermal parameters of starch in raw rice, sweet rice wine and vinasse.

Samlples	Melting temperature (°C)			ΔH (J/ g)
	T_O_	T_P_	T_C_	
IRS	73.83 ± 0.40^d^	79.68 ± 0.39^d^	84.03 ± 0.46^d^	4.07 ± 0.06^e^
GRS	64.07 ± 0.310^e^	71.59 ± 0.12^e^	79.92 ± 0.31^e^	10.63 ± 0.21^c^
IRWS	79.82 ± 1.51^c^	90.49 ± 1.48^c^	100.19 ± 1.18^c^	5.83 ± 0.40^d^
GRWS	89.32 ± 0.48^a^	101.31 ± 1.69^a^	111.81 ± 1.45^a^	12.64 ± 0.54^b^
IRVS	85.89 ± 0.27^b^	95.22 ± 0.20^b^	105.55 ± 0.72^b^	14.07 ± 0.68^a^
GRVS	74.36 ± 0.18^d^	79.56 ± 0.10^d^	85.65 ± 0.52^d^	4.54 ± 0.17^e^

A Data with different alphabets in the same column were different with statistical significance (*p* < 0.05). Mean values are given with standard deviations (*n* = 3).

### 3.9 In vitro digestibility

The digestibility of starch in vinasse and sweet rice wine were investigated, with raw rice starch and its cooked counterpart utilized as control groups. As shown in Table 4, the RS and SDS contents of GRS were 46.27 % and 25.01 %, respectively, which exceeded those of IRS (RS: 40.57 %; SDS: 23.38 %). The digestibility of raw rice starch is closely linked to its crystalline structure, with GRS exhibiting higher crystallinity than IRS, thereby conferring greater resistance to enzymatic hydrolysis (Lin et al., 2024). Gelatinization resulted in reductions of RS content from 40.57 % to 25.48 % in IRS and from 46.27 % to 16.15 % in GRS. This phenomenon is attributed to the substantial increase in digestibility caused by the disruption of crystalline structures during gelatinization (Lin et al., 2024). However, IRS demonstrated higher resistance to digestion compared to GRS, owing to its higher amylose content, which facilitated the formation of ordered structures through retrogradation (Yang et al., 2023). Following fermentation of cooked rice, the SDS and RS contents of vinasse starch was significantly enhanced. This is attributed to the preferential enzymatic hydrolysis of readily digestible starch during rice fermentation (Zhang et al., 2016). Notably, IR*V*S exhibited significantly higher RS content than GRVS. Based on the whole-molecule size distributions analysis, IRVS contained higher amylose content and long amylopectin branches, both of which readily form helical structures that impose steric hindrance on digestive enzymes (Yang, Zhong, et al., 2021). Small-molecular starch fragments in the sweet rice wine demonstrated the highest digestion resistance. Despite their limited long-range ordered structures, these fragments exhibited abundant short-range ordered configurations and were predominantly composed of long amylopectin branches and short-chain amylose, collectively forming enzyme-resistant architectures (Shi et al., 2025). Furthermore, compared to GRWS, IRWS displayed significantly elevated RS content, which correlated with its higher proportion of long linear dextrins. In summary, RDS in cooked rice starch was hydrolyzed preferfully during the fermentation process, which promoted the accumulation of SDS and RS in vinasse, among which the most resistant structure was found in fermentation mash.

**Table 4:** The RDS, SDS, and RS contents of starch in raw rice, sweet rice wine and vinasse.

Samples	RDS (%)	SDS (%)	RS (%)
IRS	36.05 ± 0.55^b^	23.38 ± 0.31^h^	40.57 ± 0.26^b^
GRS	28.72 ± 0.21^d^	25.01 ± 0.74^g^	46.27 ± 0.94^a^
Cooked IRS	30.56 ± 0.22^c^	43.96 ± 0.47^d^	25.48 ± 0.64^e^
Cooked GRS	38.59 ± 0.42^a^	45.26 ± 0.36^c^	16.15 ± 0.14^g^
IRWS	24.84 ± 0.47^f^	38.12 ± 0.49^f^	37.04 ± 0.84^c^
GRWS	26.61 ± 0.72^e^	41.54 ± 0.63^e^	31.85 ± 1.02^d^
IRVS	21.91 ± 0.26^g^	47.21 ± 0.58^b^	30.88 ± 0.65^d^
GRVS	25.35 ± 0.88^ef^	51.29 ± 0.97^a^	23.36 ± 1.81^f^

## 4 Conclusion

The multi-scale structural and digestibility of starch in sweet rice wine and vinasse were systematically investigated in this study. Molecular weight distribution and whole-molecule size distribution revealed that amylopectin, especially its short-branched chains, was preferentially utilized during fermentation. Vinasse starches exhibited enhanced digestion resistance compared to cooked rice starch, while their fundamental structural frameworks remained largely preserved. GRVS predominantly retained its original branched structure, whereas IRVS underwent significant structural alterations, including shifts in branch-chain distribution and the formation of a B + V-type crystallization due to high amylose content and starch retrogradation. Additionally, small starch granules in both indica and glutinous sweet rice wine demonstrated long linear dextrins, B + V-type crystallinity, high gelatinization temperatures and resistance to digestibility. These findings provide a foundation for developing sweet rice wine and vinasse products with optimized texture and sensory attributes. Future studies should explore the influence of starch-derived molecules on the flavor and sensory profiles of sweet rice wine.

## CRediT authorship contribution statement

**Yongqiang Gong:** Writing – original draft, Data curation. **JiaXing Hu:** Formal analysis. **Ting Xie:** Visualization. **Hongyu Mou:** Software. **Shuzhi Xiao:** Validation. **Zihan Yao:** Data curation. **Tao Yang:** Formal analysis.

## Declaration of competing interest

The authors declare that they have no known competing financial interests or personal relationships that could have appeared to influence the work reported in this paper.

## Data Availability

The data that has been used is confidential.
